# Migraine, arousal and sleep deprivation: comment on: “sleep quality, arousal and pain thresholds in migraineurs: a blinded controlled polysomnographic study”

**DOI:** 10.1186/1129-2377-14-50

**Published:** 2013-06-10

**Authors:** Catello Vollono, Elisa Testani, Anna Losurdo, Salvatore Mazza, Giacomo Della Marca

**Affiliations:** 1 Institute of Neurology, Catholic University, Policlinico Universitario ‘A. Gemelli’, Largo A. Gemelli, 8-00168, Rome, Italy

**Keywords:** Migraine, Sleep, Sleep deprivation, Cyclic alternating pattern, Arousal, Pain

## Abstract

We discuss the hypothesis proposed by Engstrom and coworkers that Migraineurs have a relative sleep deprivation, which lowers the pain threshold and predispose to attacks. Previous data indicate that Migraineurs have a reduction of Cyclic Alternating Pattern (CAP), an essential mechanism of NREM sleep regulation which allows to dump the effect of incoming disruptive stimuli, and to protect sleep. The modifications of CAP observed in Migraineurs are similar to those observed in patients with impaired arousal (narcolepsy) and after sleep deprivation. The impairment of this mechanism makes Migraineurs more vulnerable to stimuli triggering attacks during sleep, and represents part of a more general vulnerability to incoming stimuli.

## 

Sir,

We read with great interest the paper by Engstrøm et al. (2013) “*Sleep quality, arousal and pain thresholds in migraineurs: a blinded controlled polysomnographic study*” [1]. We appreciated the methodology of the study, nevertheless we believe that the hypothesis formulated by the Authors in their conclusion deserves further comments.

## Findings

1. In their study, the Authors evaluated psychophysiological measures and polisomnography (PSG) parameters in Migraineurs and healthy volunteers; in discussing their results, the Authors suggested the hypothesis that Migraineurs have a relative sleep deprivation, which in turns lowers the pain threshold and predispose to headache attacks [1]. This hypothesis is theoretically conceivable, since it is based on solid data coming from Migraine and sleep literature. It is well known, in fact, that sleep deprivation may enhance the response to pain stimuli [2]; moreover, sleep restriction is one of the most common triggers of migraine attacks [3].

2. Nevertheless, other clinical and neurophysiological observations are not in agreement with the Authors’ hypothesis. In fact, some data indicate that it is sleep continuity disturbance, rather than simple sleep restriction, that impairs endogenous pain-inhibitory function and increases spontaneous pain [4]. Moreover, it is likely that modification of routinely sleep habits is the major trigger, since increased sleep duration predisposes to Migraine attacks to the same extent as sleep deprivation [3].

3. The results of subjective (logs) and objective (PSG) sleep analysis reported by Engstrøm et al. (2013) [1] do not seem to be fully consistent with their hypothesis that ‘*migraineurs on the average suffer from a relative sleep deprivation and need more sleep than healthy controls*’. In fact, in their paper, Migraineurs showed an average total sleep time slightly longer than controls, even though no statistically significant differences were observed between Migraineurs and Controls in sleep macrostructure. Even the analysis of the sleep logs does not support the deprivation hypothesis, since Migraineurs and controls had similar average sleep time.

4. Conversely, the Authors observed in Migraineurs a decrease of fast arousal index, associated with an increase of awakenings. This finding, apparently contradictory, is similar to that reported in a previous study of our group [5]. In that paper [5] we studied a selected population of sleep-related Migraineurs and we observed a reduction of the Cyclic Alternating Pattern (CAP). Therefore, we suggested that the slow-frequency, high-amplitude arousal fluctuations described in the CAP model are also essential to dump the effect of incoming disruptive stimuli, and to protect sleep from external perturbations. The impairment of this mechanism makes Migraineurs more vulnerable to stimuli triggering attacks during sleep.

## Discussion: alternative hypothesis

On the other hand, CAP modifications similar to those described in Migraineurs have been reported in normal subjects following sleep deprivation. Poryazova et al. (2011) [6] investigated the regulation NREM sleep in normal subject and narcolepsy / cataplexy patients before and after sleep deprivation. This study [6] demonstrated that: 1) patients with impaired arousability (narcolepsy patients) have lower CAP rate that controls; and 2) sleep deprivation induces a reduction of CAP rate, which, in normal subjects after deprivation, reached values similar to those reported in our study for Migraineurs.

Whatever the interpretation, we believe that these data, taken together, suggest that Migraine is a condition in which an impairment of arousal responses occurs. As suggested by Borsook et al. (2012), Migraine is associated with alterations in normal homeostatic mechanisms (e.g., altered sleep, abnormal autonomic function). When stressors become frequent and/or severe, allostatic responses may be dysregulated and become maladaptive (“allostatic load”). Such allostatic load, in turn, may alter brain responses to stressors; therefore, behavior and systemic physiology are altered in ways that can, in a vicious cycle, lead to further allostatic load [7]. In this sense, arousals during sleep must be considered as an homeostatic, adaptive response, rather than a ‘pathologic’, disruptive phenomenon, which can be detrimental for sleep. The impaired arousability during sleep, seen in this view, is part of a more general decreased ability of Migraineurs to process incoming stimuli.

## Abbreviations

CAP: Cyclic alternating pattern; NREM: Non rapid eye movement; PSG: Polysomnography.

## Competing interests

The authors declare that they have no competing interests.

## Authors’ contributions

CV: data analysis and interpretation, manuscript drafting and revision, final approval of the version to be published. ET: data analysis and interpretation, manuscript drafting and revision, final approval of the version to be published. AL: data analysis and interpretation, manuscript drafting and revision, final approval of the version to be published. SM: data analysis and interpretation, manuscript drafting and revision, final approval of the version to be published. GDM: data analysis and interpretation, manuscript drafting and revision, final approval of the version to be published. All authors read and approved the final manuscript.
